# Pharmacogenomics and its Role in Cardiovascular Diseases: A Narrative Literature Review

**DOI:** 10.2174/011573403X334668241227074314

**Published:** 2025-01-31

**Authors:** Maryam Kayani, Gadde Krishna Sangeetha, Swapna Sarangi, Leela Sarmada Gaddamanugu, Shelja Sharma, Victor O. Adedara, Saria Abdallah, Kristina Katz, Glendalys Rodríguez Mora, Sravani Kommuru, Zahra Nazir

**Affiliations:** 1 Shifa College of Medicine, NCBMS Tower, Sector H8/4, Islamabad, 46000, Pakistan;; 2 Mediciti Institute of Medical Sciences, Ghanpur, Telangana, India;; 3 Lady Hardinge Medical College and Associated Hospitals, Shaheed Bhagat Singh Marg, Connaught Place, New Delhi 110001, India;; 4 Mysore Medical College and Research Institute, Irwin Road, Mysore, Karnataka, 570001, India;; 5 CMHO Office, Jaipur 1st, Sethi Colony, Raja Park, Jaipur, Rajasthan, India;; 6 School of Medicine, St George’s University True Blue, Grenada, West Indies;; 7 Beirut Arab University, Beirut Campus, Tarik El Jadida, Riad El Solh, Beirut, 11072809, Lebanon;; 8 Carleton University, 1125 Colonel By Dr, Ottawa, Ontario, Canada;; 9 Universidad Catolica Nordestana (UCNE), Sección Los Arroyos, San Francisco de Macorís, Provincia Duarte, República Dominicana;; 10 Pinnamaneni Siddhartha Institute of Medical Sciences and Research Foundation, Chinna Avutapalli, Gannavaram, Vijayawada, 521101, Krishna (Dt), Andhra Pradesh, India;; 11 Baqai Medical University, Karachi, Pakistan

**Keywords:** Statins, calcium channel blockers, arrhythmias, pharmacogenomics, cardiovascular diseases, genetic polymorphisms

## Abstract

Pharmacogenomics has transformed the way we approach the treatment of the most common diseases worldwide, especially cardiovascular. In this article, we highlight the main categories of drugs involved in major cardiovascular diseases (CVD), related genetic variability and their effects on metabolism in each case of contrastive operability. This not only explains disparities in treatment outcomes but also unfolds customised management based on genomic studies to improve efficiency and limit side effects. Genetic variations have been identified that impact the efficacy, safety, and adverse effects of drugs commonly used in the treatment of CVD, such as Angiotensin converting Enzyme Inhibitor (ACEI), Angiotensin Receptor Blocker (ARBs), calcium channel blockers, antiplatelet agents, diuretics, statins, beta-blockers, and anticoagulants. It discusses the impact of genetic polymorphisms on drug metabolism, efficacy, and adverse reactions, highlighting the importance of genetic testing in optimizing treatment outcomes. Pharmacogenomics holds immense potential for revolutionizing the management of CVD by enabling personalized medicine approaches tailored to individual genetic profiles. However, challenges such as clinical implementation, cost-effectiveness, and ethical considerations need to be addressed to completely incorporate pharmacogenomic testing into standard clinical practice. Continued research and clinical diligence are required for the utilization of pharmacogenomics to improve therapeutic outcomes and reduce the burden of CVD globally.

## 1 INTRODUCTION

The human genome is the largest and most complex genome to be sequenced [1]. In 510 BCE, Pythagoras noticed that certain people who eat fava beans became ill, which is now formally known as a clinical manifestation G6PD deficiency. In the modern days, certain drugs are avoided based on G6PD deficiency, hence, it is a good example of the utility of pharmacogenomics [2]. Pharmacogenomics seeks to maximize therapeutic outcomes while minimizing side effects by analysing genetic differences that impact drug metabolism, effectiveness, and toxicity [3].

Cardiovascular diseases are on the rise towards one of the most common causes of death worldwide, contributing to about 17.9 million deaths per year, which is about 32% of all global deaths [4]. Even with improvements in therapeutic approaches, patient responses to cardiovascular drugs might differ substantially, which can result in ineffectiveness, unfavourable side effects, and treatment failures. Pharmacogenomics has proven to be quite useful in the treatment of cardiovascular diseases [5]. The safety and efficacy of many drugs such as anticoagulants (warfarin), antiplatelets (clopidogrel), statins, beta-blockers, anti-arrhythmic and anti-hypertensives are metabolised by CYP (cytochrome P450) enzyme type and activity. The CYP enzymes are encoded by specific genes *e.g.*, *CYP2C9* and *CYP3A4.* Hence, determining the genetic variations in the genes encoding these enzymes plays a pivotal role in the management of cardiovascular diseases [6].

The cost of genome sequencing has been declining exponentially over the past decade; therefore, it will become more accessible and affordable to the general population in the near future [7]. The CPIC (Clinical Pharmacogenetics Implementation Consortium) guidelines included the gene-drug combinations along with appropriate dosing recommendations to guide the physician regarding the use of different cardiovascular drugs [8]. The application of pharmacogenomics in cardiovascular medicine has been one of the most explored aspects of pharmacogenomics. Yet, there seems to be a gap in the application of this database in the clinical setting due to limited training and exposure to pharmacogenomics and its application. In addition, the awareness regarding the provision of pharmacogenomics and the credibility of its explication is equally paramount [9].

This study explores how genome-wide sequencing has transformed the approach to the treatment of cardiovascular diseases. We aim to investigate the significance of pharmacogenomics in CVDs, assessing possible applications, challenges, and future directions by summarizing the available data and highlighting significant research and developments in the field.

### 1.1 Key Points

1. Many studies have shown the pharmacogenomic importance of ACE, Angiotensinogen (AGT), NOS3 (bradykinin pathway) for ACEi and ARB responses; *CYP2D6, ADRB1, ADRB2, GRK5* for β blockers responses and *CES 1, CES2, and ABCB 1, CYP3A4* for newer anticoagulants.
2. For antiplatelets such as clopidogrel and ticagrelor, the type of metabolizers *i.e.* normal, intermediate or poor dictate the use of clopidogrel compared with ticagrelor or prasugrel. Warfarin metabolism and its dosage is mainly guided by variation in *CYP2C9* function.
3. Polymorphisms in *GNB2, ANP, ACE, ADD1* influences the effectiveness of diuretics and polymorphisms in *CACNA1C, CYP3A4, CYP3A5* influences the effectiveness of calcium channel blockers.
4. Among the antiarrhythmics, response to flecainide therapy has been linked to genetic variations in *SCN5A* gene, whereas sotalol and dofetalide are dependent on *KCNH2* gene variation. Regarding statins, the concentration of the drug is found to be dependent on variations in *SLCO1B1* gene, while the efficacy of statins is increased by presence of 2677T allele.
5. The challenges and limitations of cardiovascular pharmacogenomics indicates the need for more evidence and research to achieve maximum effectiveness and safe clinical application.

## 2 MAIN TEXT

Drug response varies significantly amongst patients, with a large portion of this variability having a hereditary foundation. A patient's higher risk of side effects or potential benefit from different treatments or dose adjustments can be identified through genetic testing. Therefore, it is possible to maximize the effectiveness of medication and enhance patient outcomes by using pharmacogenomic insights [10].

Recent literature highlights the significant influence of genetic variations on pharmacological efficacy and metabolism [11]. Genetic variation can influence how the body reacts to medicines through a variety of pathways, including genes involved in the pathophysiology of disease development, changes to how drugs interact with genes, and polymorphisms in genes linked to drug transporters. Multiple genetic variants encoding drug-metabolizing enzymes and metabolic processes can also impact drug responses [12]. Pharmacogenomics is the study of genetic variation in targets, transporters, receptors, and enzymes that metabolize drugs, as well as how these genetic variants interact to generate phenotypes related to drugs, such as toxicity or responsiveness [13]. The pharmacokinetics and pharmacodynamics of drugs that are frequently used to treat CVD can be strongly impacted by these genetic variations [14]. Cardiovascular disorders (CVDs) are managed with a wide range of pharmacological agents. Drugs influenced by the most prevalent gene polymorphisms are listed below.

### 2.1 ACEI & ARBs

ACE inhibitors are advised as first-line antihypertensive medication in guidelines published by the European Society of Cardiology (ESC) and the American Heart Association/American College of Cardiology (AHA/ACC) [15]. Several cardiovascular diseases, including heart failure, stroke, and coronary disease, are significantly increased by hypertension, which is treated and managed with ACE inhibitors [16]. The class of drugs known as angiotensin receptor blockers, or ARBs, is also used to treat hypertension, congestive heart failure, and diabetic nephropathy. They are frequently used to substitute ACE inhibitor medication in patients who cannot tolerate them due to a chronic cough brought on as a side effect of ACEI [17].

#### 2.1.1 Mechanism of Action

Angiotensin-converting enzyme, or ACE inhibitor, prevents the conversion of angiotensin I to angiotensin II. Reduced angiotensin II synthesis improves natriuresis, decreases blood pressure, and inhibits cardiac myocytes and smooth muscle from remodeling. Preload and afterload are also decreased by decreased venous and arterial pressure [18]. The balance between the vasoconstrictive and salt-retentive characteristics of Angiotensin II and the vasodilatory and natriuretic features of Bradykinin is regulated by the angiotensin-converting enzyme. By reducing the synthesis of angiotensin II and the breakdown of bradykinin, ACE inhibitors upset this equilibrium [19]. Similar mechanisms are used by ARBs to control blood pressure. ARBs counteract AII's effects on AT1 receptors [20]. Fig. (**1**) outlines the mechanisms of action of ACEI and ARBs [21] and the genes involved in these processes [22].

#### 2.1.2 Pharmacogenomics

The efficacy and acceptability of ACE inhibitors vary significantly despite these known mechanisms of action. Given that the renin-angiotensin-aldosterone system has a high probability of inheritance [23], studies have suggested that genetic markers could be helpful in identifying people who are most likely to tolerate or benefit from these medications. The genes involved in the renin-angiotensin system and the bradykinin pathway are the most plausible candidate genes for a pharmacogenomic approach to these medications, as shown by the above-mentioned figure. Multiple pleomorphisms have been investigated in intron 16 of ACE [24], one of the aforementioned genes in RAAS (Renin-Angiotensin-Aldosterone System). Indeed, in Asian and Caucasian populations, the D allele of this polymorphism appears to be linked to greater levels of ACE compared to in African and Americans [25]. The effects of ACE inhibitors vary because some studies indicate that the D allele is associated with better hypertensive control, while other literature indicates that the I allele is superior [26].

*AGT* is another gene that is involved in the renin-angiotensin system and may, therefore have an impact on how the body reacts to ACE inhibitors and ARBs. Angiotensinogen is encoded by the *AGT* gene, and the *SNP Met235Thr (rs699)* is the most researched variation of this gene [27]. However, in a study conducted by Konoshita *et al.* [28], *AGT* gene polymorphisms do not significantly impact the way ACEI or ARBs regulate blood pressure [28]. It has been demonstrated that genetic polymorphisms in the *ACE* and *AGT* genes, specifically *M235T* and *T174M* in *AGT* and *ACE* I/D in ACE, affect the plasma levels of these enzymes. For instance, the TT allele of the *M235*T polymorphism leads to increased levels of angiotensinogen [29].

The final step of aldosterone synthesis in juxtaglomerular cells is catalyzed by the enzyme aldosterone synthase, which is encoded by the *CYP11b2* gene, as demonstrated by Freel *et al.*, in the MRC BRIGHT study [30]. This gene contains a significant variant called *SNP −344C/T (rs17998)*, which has been linked to altered blood pressure responses, susceptibility to hypertension, and aldosterone levels [31]. Although the C allele of this *SNP* was linked to this effect [32], the T allele was linked to the same effect in SILVHIA trial [33].

Studies have suggested that the *AGTR1* gene is a key genetic factor that determines how effectively ACE inhibitors and angiotensin II receptor blockers treat heart failure [34] and hypertension [35].

By detecting potential heterogeneities in treatment effects and directing therapy to those most likely to benefit, predicting which patients will benefit or forfeit their benefits from ACE-inhibitor therapy can improve efficiency and cost-effectiveness. The EUROPA trial [36] was conducted to evaluate if perindopril, an ACE inhibitor, decreased the risk of adverse cardiovascular events. Cardiovascular mortality, myocardial infarction, or cardiac arrest were the main endpoints, and 12218 patients were enrolled with a mean follow-up period of 4.2 years. Perindopril can dramatically improve prognosis in people with stable coronary heart disease who do not have heart failure. But in this trial, 50 patients had to take perindopril for four years, or 200 patient years, in order to avoid one significant cardiovascular incident [36]. Brugts *et al.* [37] presented data from the Perindopril Genetic association study (PERGENE), a sub-study of the randomized, placebo control (EUROPA) trial. In patients with stable CAD, the PERGENE trial showed a pharmacogenetic risk profile that predicted the perindopril ACE inhibitor's therapeutic efficacy. Patients with relative resistance to ACE medications were shown to have unfavourable alleles of genetic variations in the *AT1* and *BK1* receptor genes. According to the results of PERGENE [37], three of the four patients with stable CAD who took part in EUROPA benefited more from ACE inhibitor medication, while the non-responders benefited from perindopril treatment considerably less. Relative risk reduction was +26% and 33%, respectively, in groups of patients with ≥3 and <3 unfavourable alleles. It was suggested that in patients with more unfavorable alleles, discontinuing perindopril treatment could prove to be cost-effective while improving medicine’s overall effectiveness [37].

Numerous studies have demonstrated the pharmacogenomic importance of *NOS3* for ACEi and ARB responses [38]. The pleiotropic effects, such as those arising from vasodilation by nitric oxide (NO) produced by the endothelial NO synthase (*NOS3*), are linked to the beneficial effects of ARB [39]. Mason *et al.* [40] found that in contrast to heterozygous cells, endothelial cells homozygous for the C allele (the −786T/C polymorphism in *NOS3, rs2070744)* have a greater ability to produce NO when treated with ARB olmesartan. The authors came to the conclusion that individuals with hypertension who carried the C allele could respond more favorably to olmesartan and enalapril [40]. Furthermore, the T allele for the *NOS3 −665C/T SNP (rs3918226)* has been linked to increased enalapril responses, according to Oliveira-Paula *et al.* [41].

Silva *et al.* [42] conducted a study of gene–gene interactions, which showed that a better response to enalapril medication is linked to the coexistence of the TC genotype for the *rs2070744 SNP* in the promoter region of the *NOS3* gene and the CC genotype for the *rs1799722 SNP* in the *BDKRB2* gene [42]. Oliveira-Paula *et al.* [41] conducted a combined analysis o*f PRKCA, BDKRB2,* and *NOS3* and found that the interaction described above was statistically significant when the *rs16960228 SNP* in the *PRKCA* gene was present in the GG genotype. This observation could be linked to PKCα's enhancement of *NOS3* gene transcription, upregulation of eNOS activity, and consequent promotion of enhanced NO generation and vasodilation [41]. It concludes that the responses to *ACE* inhibitors and ARB are modulated by the presence of pleomorphisms in different genes which is summarized in Table **1**.

### 2.2 Beta Blockers

Beta blockers are widely used drugs in cardiovascular diseases. They are known to decrease mortality in cardiovascular events related to hypertension. Cardiomyocytes express beta-adrenergic receptors, which are triggered by catecholamines in the bloodstream or noradrenaline produced by sympathetic synapses. The G protein-coupled receptors (GPCRs) family has three subtypes of their corresponding receptors: Beta1, Beta2, and Beta3. Numerous physiological processes, such as heart contraction and the release of renin from the kidney's juxtaglomerular cells, are brought on by the activation of Beta1-adrenergic receptors, as depicted by Fig. (**2**) [43].

Although β-blockers are widely used, the current data indicates that hypertension treatment with beta-blockers results in slight decreases in cardiovascular disease (CVD) and has minimal to no impact on mortality rates. Several studies indicate that *ACE* inhibitors and thiazides are superior to β-blockers in the treatment of hypertension and β-blockers failed as monotherapy for hypertension in 30-60% of patients [44, 45]. This raises the suspicion of genetic variability affecting the response to β-blockers. The diversity within genes impacting their pharmacokinetics and pharmacodynamics leads to significant differences in how patients respond to them. The current focus on precision medicine offers a chance to review existing research on the pharmacogenomics of β-blocker treatment and assess whether it's suitable to apply these genetic findings in clinical settings [46]. The genes that exhibit the most compelling evidence of influencing responses to β-blocker therapy are *CYP2D6, ADRB1, ADRB2,* and *GRK5.*

#### 2.2.1 CYP2D6

Cytochrome P450 2D6 (*CYP2D6*) is responsible for the metabolism of several β-blockers, including Carvedilol, metoprolol, Nebivolol, Propranolol and Timolol [45]. Others, such as Atenolol and Bisoprolol, are poorly metabolized by *CYP2D6* [5].

Pharmacokinetic characteristics (*e.g.*, half-life, oral clearance, area under the plasma concentration-time curve) are directly linked to *CYP2D6* genotype and vary between rapid, intermediate and poor metabolizers [46]. According to a study targeting the association between the pharmacokinetics of metoprolol and *CYP2D6* genotype, metoprolol oral clearance was almost 6-fold lower in PMs (poor metabolizers) than in NMs (normal metabolizers). As compared to NMs, metoprolol oral clearance was 1.5 times lower in IMs bur 2.6 times greater in UMs (ultra-rapid metabolizers) [46].

In addition, compared to extensive metabolizers, poor metabolizers had a 66% lower clearance of R-carvedilol and a 156% greater area under the plasma concentration-time curve. Response to β-blockers also exhibit variations among ethnicity, where 5 to 10% of Caucasians exhibit a poor metabolism by *CYP2D6* in contrast to 1% only of Asians and 2 to 3% of African Americans. In terms of clinical practice, this could result in reduced dosage needs to attain optimal β-blockade and, subsequently, less adverse effects. In this context, a study explained that the risk of pulmonary adverse effects with B1-selective drug in a patient suffering from asthma is much more pronounced in poor metabolizers. Likewise, poor metabolizers treated with carvedilol or metoprolol for heart failure have a higher risk of decompensation [45]. Furthermore, several studies suggested that the degree of β-blockage and HR response may vary according to *CYP2D6* activity. Particularly, *CYP2D6* was identified in one study as a key factor influencing the variation in the HR response to metoprolol [46]. A decreased HR with β-blockers is much more pronounced with IMs and specially in PMs than EMs. While not all studies have documented this finding, it’s noteworthy that some have found a higher incidence of asymptomatic bradycardia (HR <60 b/min) in patients with poor metabolism of drugs by *CYP2D6* [5]. Most variations in *CYP2D6* genotype were shown to have a main effect on HR response to β-blockers rather than hypertension, where its implication remains unclear [47].

#### 2.2.2 ADRB1, ADRB2 and GRK

Besides CYP2D6 genotype, three other genes -*ADRB1, ADRB2,* and *GRK5*— present an area of interest in the pharmacokinetics of β-blockers. β1 and β2 adrenergic receptors are encoded by *ADRB1* and *ADRB2* respectively. *Ser49Gly (rs1801252)* and *Arg389Gly (rs1801253)* are the most common variations in *ADRB1* [8]. A significant improvement in LVEF has been demonstrated in patients on β-blockers who express the *389Arg* genotype when compared to those with *Gly* genotype. Additionally, the *389Arg* genotype was shown to be correlated with a greater decrease in blood pressure and heart rate and most importantly, a drop in hospitalization and mortality rates [48]. One study suggested that *ADRB1* affects mainly DBP but not SBP in hypertensive patients treated with β-blockers [46]. In contrast to *ADRB1*, studies done on ADRB2 polymorphisms were inconclusive and *ADRB2* was not shown to affect β-blockers response. It has been mostly linked to *Gly16Arg* and *Gln27Glu,* which exhibit some decrease in heart rate and blood pressure in patients treated with β blockers. This may be important in future research and more studies are needed to be done to demonstrate a correlation between these genotypes’ variations and response to β blockers [8]. Another important gene variation is related to G protein-coupled receptor kinase 5 (GRK-5), which is responsible for desensitization of G protein-coupled receptors, including β-adrenergic receptors, by promoting receptor down-regulation once they are occupied by ligands. The *GRK5* that encodes Leu41 was found to intensify the desensitization process of the β-1 adrenergic receptor in laboratory settings, thus diminishing the receptor stimulation and cAMP production. It’s noteworthy that improved outcomes in HF (heart Failure) and HTN (hypertension) in association with *GRK5* were independent of β blocker treatment in most but not all studies, which indicates that it’s more related to the underlying physiology of the cardiovascular disease itself rather than a drug effect [46].

Nonetheless, more research should be done to implement pharmacogenomics of β blockers in clinical practice and take advantage of the highly important variations among different genotypes to enhance the benefit of these drugs.

### 2.3 Clopidogrel

Treatment with clopidogrel, an anti-platelet agent in patients with acute coronary syndrome, following or undergoing percutaneous coronary intervention (PCI), is most commonly and widely used to prevent the risk of myocardial infarction and stroke in these patients. Clopidogrel is a thienopyridine prodrug that bio-transforms in the liver to an active metabolite, irreversibly inhibiting purinergic P2Y12 receptor and platelet activation [49].

After ingesting orally, clopidogrel is absorbed in the duodenum actively by the P-glycoprotein efflux pump coded by the *ABCB1* gene [50]. About 85% of the drug absorbed is hydrolyzed into an inactive carboxylic acid derivative by carboxylesterase-1 (CES1). The remaining 15% of the absorbed drug is converted to an active thin metabolite by two sequential oxidative steps. Many CYP450 enzymes (*CYP1A2, CYP2B6, CYP2C9, CYP2C19, CYP3A4/5*) participate in these steps, particularly *CYP2C19*. Firstly, clopidogrel is oxidized to 2-oxo-clopidogrel (intermediate metabolite). Then 2-oxo-clopidogrel undergoes two pathways: hydrolyzed to an inactive acid metabolite by *CES1* or oxidized to an active thiol metabolite. The active metabolite binds to the P2Y12 purinergic receptors upon entering the systemic circulation and, thus inhibiting ADP-mediated platelet activation and aggregation [50].

#### 2.3.1 Pharmacogenomics

It was in the late 1990s when clopidogrel was recognized as an anti-platelet drug, but the role of *CYP2C19* in activating clopidogrel to an active metabolite was established in 2006 [51]. The gene encoding *CYP2C19* is vastly polymorphic with more than 35 star alleles according to the pharmacogene variation consortium. *CYP2C19* alleles are divided based on the allele function into allele with no function (*2,*3), allele with decreased function (*9), allele with normal function (*1), and allele with increased function (*17) [2]. Based on allele function influencing the plasma concentration of clopidogrel active metabolite, the individuals are classified into groups such as individuals with two normal function alleles(*1/*1) are normal metabolizers (NMs), individuals with two non-functional alleles (*2/*2,*3/*3,*2/*3) are poor metabolizers (PMs), individuals with one normal function allele and one no function allele (*1/*2,*1/*3) or with one no function allele and one increased function allele (*2/*17,*3/*17) are intermediate metabolizers, individuals with one normal function allele and one increased function allele (*1/*17) are extensive metabolizers, individuals with two increased function alleles (*17/*17) are ultra-rapid metabolizers. There is reduced clopidogrel active metabolite in *CYP2C19* IMs and PMs when compared to PMs, according to various studies [52].

The occurrence of the no function variant CYP2C19*2 is greater among Asians than Caucasians and the occurrence of another no function variant CYP2C19*3 is almost absent in Africans to 15% in East Asians [51]. CYP2C19*2 is associated with reduced clopidogrel activation, reduced antiplatelet function and a higher risk of adverse cardiac events. As described in a large meta-analysis, the carriers of CYP2C19*2 variant have a greater of developing major cardiovascular events like cardiovascular death, myocardial infarction, stroke, and in-stent thrombosis [53].

POPular-Genetics is a randomized controlled, assessor-blinded trial. The trial population consisted of patients having signs and symptoms of STEMI FOR 30 minutes to 12 hours and who have undergone PCI with stent implantation. The patients were either given P2Y12 inhibitors based on early *CYP2C19* genetic testing (genotype guided group) or were treated with ticagrelor or prasugrel (standard treatment) in 1:1 ratio. The results were that death from major cardiovascular events occurred in 5.1% of genotype guided group and 5.9% in the standard treatment group at 12 months. The findings in this trial indicate that there is no greater risk of combined death in patients without *CYP2C19* no -function allele (genotype guided group) receiving clopidogrel than those who were treated with ticagrelor or prasugrel (standard treatment) [54].

A randomized control trial such as TAILOR-PCI consisted of patients who have acute coronary syndrome (ACS) or stable coronary artery disease (CAD). *CYP2C19* LOF carriers were given ticagrelor and non-carriers clopidogrel based on genotyping (genotype guided group) and patients in conventional group were given clopidogrel. The results indicated that end point cardiovascular death occurred in 4.0% in genotype-guided therapy patients and in 5.9% in conventional group, thus, showing there is no major difference in end point cardiovascular death between genotype guided therapy and conventional therapy [55].

A study by Hulot *et al.,* is a multisite observational study. The participants in this study are patients presenting with STEMI within 24 hours and are scheduled for PCI. 12-month outcomes of patients with wild-type genotype or gain of function allele (class 1) and patients with loss of function allele who were treated with thienopyridine (class 2) are studied in this study. The endpoint in class 1 was observed in 3.31% percent and the endpoint in class 2 was observed in 3.04%. Thus, there is no significant difference between class 1 and class 2. However, much worse outcomes were observed in carriers of LOF alleles without treatment adjustment (15.6%) [56].

Pharmaclo, a randomized control trial studied by Notarangelo *et al.* [9], assessed whether there would be better clinical results on selecting anti platelet therapy based on patients' genetic and clinical features than with standard care, which is solely based on clinical features. Ticagrelor was used more frequently in the pharmacogenomic arm and clopidogrel more frequently in the standard care arm. In 15.9% of patients belonging to the pharmacogenomic arm, the primary end point occurred, while in 25.9% of standard of care arm observed primary endpoint. This draws the conclusion that there were reduced ischemic and bleeding events in patients with ACS when a personalized approach was taken in selecting anti platelet therapy [57].

The label of clopidogrel was added with a boxed warning regarding decreased clopidogrel activity in poor metabolizers by the FDA in 2010. Both IMs and PMs are addressed mainly focusing on patients' ACS and PCI in CPIC guidelines for clopidogrel use [10]. In ultra-rapid metabolizers (increase in clopidogrel active metabolite, low platelet reactivity treatment with low bleeding risk), rapid metabolizers (increase in or normal clopidogrel active, low platelet reactivity treatment with low bleeding risk), normal metabolizer (normal clopidogrel active metabolite, normal platelet reactivity treatment) there is strong recommendation to use standard dose (75 mg/day) of clopidogrel. In intermediate metabolizers (decreased clopidogrel active metabolite, higher on platelet reactivity treatment with greater risk of adverse cardiac and cerebrovascular events) and poor metabolizers (marked decrease in clopidogrel active of metabolite, higher platelet reactivity treatment with higher risk for cardiac and cerebrovascular events), there is a strong recommendation to avoid clopidogrel and use ticagrelor or prasugrel at standard dose provided no other contraindications [49].

Testing for *CYP2C19* varies in different sites. At some sites, rapid genotyping is available, and the results are obtained soon after sample collection, whereas in sites where there is no possibility of rapid genotyping, the results are obtained within a week so that if patients being treated with clopidogrel have no-function allele they can be shifted to alternate therapy. However, this seperates patients who underwent PCI at greater risk of MACE. Instead, the patients can be treated with ticagrelor or prasugrel until the results of genotyping are received when rapid genotyping is not available. When the results are obtained, prasugrel or ticagrelor can be continued in IMs and PMs but in patients without no-function allele, the treatment can be shifted to clopidogrel. This approach is termed as a de-escalation approach, where clinical outcomes can be maximized, and bleeding risk can be reduced [8].

### 2.4 Anticoagulants

Historically recognized as a key factor for the synthesis of blood clotting factors in the liver, the most well-known function of vitamin K is as a cofactor for the γ-glutamyl carboxylase (GGCX) enzyme responsible for the post-translational modification of vitamin K-dependent proteins (VKDPs) through the conversion of specific glutamic acid (Glu) into calcium binding γ-carboxyglutamic acid (Gla) residues. The hepatic group of VKDPs synthesized in the liver are essential for regulating blood coagulation and comprise the coagulation factors II, VII, IX, and X, and the anti-coagulation proteins C and S [58]. Vitamin K includes two primary forms: phylloquinone (vitamin K1, VK1) and menaquinones (vitamin K2, VK2). VK1 is exclusively found in plants, while VK2 (menaquinone-5 - menaquinone-13, MK5–MK13) is produced by a series of congeners synthesized by gram-positive bacteria in the human gastrointestinal tract. Menaquinone-4 (MK4) is endogenously synthesized from VK1 in mammals and is found in animal products [59]. Fig. (**3**) demonstrates the mechanism of action of vitamin K.

Approved indications of Vitamin K administration are not extensive. Vitamin K is indicated for the prevention of hemorrhagic disease in the newborn or as an antidote to correct Vitamin K antagonist overshooting or poisoning since Vitamin K antagonists are also used as rodenticides [60, 61]. Warfarin is an oral vitamin K antagonist used for venous thromboembolism prophylaxis and prevention of stroke in atrial fibrillation. Warfarin is metabolized *via* the cytochrome P450 system by *CYP 2C9, 1A2*, and *3A4*. It is a racemic mixture, with the S-enantiomer being 2.7 to 3.8 times more potent than the R-enantiomer [62]. The effect of warfarin can be monitored by prothrombin time (PT) and international normalized ratio (INR). The mechanism of warfarin is demonstrated in Fig. (**4**).

Atrial fibrillation (AF) is the most common cardiac arrhythmia causing irregular and often abnormally fast heart rate. Patients with AF have an increased risk of heart failure, stroke, dementia, and death [63]. Cardiac remodeling, particularly of atria, results in structural and electrical changes that eventually become the cause of deranged rhythm in AF. Structural remodeling is caused by the changes in myocytes and the extracellular matrix, and fibrous tissue deposition also plays a major role in some etiologies [64].

Venous thromboembolism (VTE) includes both deep vein thrombosis (DVT) and pulmonary embolism (PE) [65]. There are several genetic conditions known to increase the risk of VTE, including factor V Leiden, prothrombin gene mutation (*G20210-A*), antithrombin deficiency, protein C deficiency, and protein S deficiency [66].

Direct oral anticoagulant (DOACS) Dabigatran is a reversible competitive inhibitor of thrombin that specifically inhibits both free and clot bound thrombin and thrombin induced platelet activation [67], It is used in venous thromboembolism, atrial fibrillation and can be used in heparin induced thrombocytopenia. Dabigatran is metabolized *via* conjugation into four acyl glucuronides, each of which is a direct thrombin inhibitor with less than 10% of the activity of the parent compound. Dabigatran neither is metabolized by nor induces any cytochrome P450 subtype [68].

Apixaban and rivaroxaban inhibit factor Xa, which are used as treatment and prophylaxis for pulmonary embolism and deep vein thrombosis, as well as prophylaxis in patients with atrial fibrillation. Apixaban is metabolized in part by *CYP3A4*; it is partly eliminated by the kidneys (25%) and, to some extent, also processed *via* CYP-independent mechanisms in the liver [69], although Rivaroxaban is metabolized by several cytochrome P450 enzymes (*CYP 3A4/5, CYP2J*2) and CYP-independent mechanisms [70], has a dual mode of elimination. Approximately two-thirds of the administered dose undergoes metabolic degradation, with half eliminated renally and the remainder by the hepatobiliary route. The final one-third of the administered dose is eliminated *via* direct renal excretion as the unchanged active substance in the urine, mainly *via* active renal secretion [71].

#### 2.4.1 Genetic Variations, Drug Response, Studies Conducted, and Recent Advances Warfarin

Warfarin is still being used today even with the discovery of newer anticoagulants in situations where there is a contraindication or not sufficient evidence for newer drugs like prosthetic heart valves, conditions like antiphospholipid syndrome, and conditions like hepatic failure [72]. Warfarin is also being used in places where a fixed dose would not be justified, for example, extreme body weight and interaction with other drugs [73].

Hence, it is imperative to understand genetic variability and the drug responses to the said variability to improve patient care. There are mainly two genes that are responsible for the metabolism of warfarin *CYP2C9* and *VOKRC1,* which are cytochrome p450 and Vitamin K epoxide reductase complex 1, respectively [47].

Although there are multiple variants of the *CYP2C9* gene, two of them stand out to influence the metabolism of warfarin, they are CYP2C9*2 CYP2C9*3 allelic variants [74]. The presence of CYP2C9*2, CYP2C9*3 and VOKRC1 genes causes over-anticoagulation [74]. It is also worth noting that different ethnicities also have different variations, for example, the above-mentioned CYP2C9*2, CYP2C9*3 and VOKRC*4 were more commonly found in the Eurasian population, while CYP2C9*8, CYP2C9*5, CYP2C9*6 and CYP2C9*11 were commonly found more among the Africans [75, 76]. Multiple studies have been conducted to prove the relation between the genetic variations and the drug response. Schwarz *et al.* [77] showed that there was a variation in the initial time taken to reach the therapeutic INR [77]. Limdi *et al.* [78] conducted a study that showed a 5.3 times higher risk of major bleeding in patients on warfarin therapy who have a variation in the *CYP2C9* gene [79]. More recent studies are focused on including more diverse ethnic populations in the pharmacogenomic studies of warfarin [76, 79].

#### 2.4.2 Newer DOACs Genetic Variation and Drug Response

Newer DOACs like Dabigatran (direct factor IIa inhibitor), Rivaroxaban, and apixaban (factor Xa inhibitors) are preferred in recent times due to the availability of fixed dosing schedules of these drugs and avoiding the regular monitoring of the patient’s coagulation *i.e.*, INR [67, 73]. Since these are a relatively newer class of drugs, studies on pharmacogenomics are still going on. It has been shown that *CES 1, CES2*, *ABCB 1,* and *CYP3A4* genes are responsible for any variations in metabolism and variation in drug response. The variations of these genes mainly occur as single nucleotide polymorphisms (SNPs) [67, 70, 80]. For dabigatran variation of *rs71647871, rs8192935 SNPs* in the *CES1* gene will lead to a decrease in peak level. Whereas, the *ABCB1* gene change in *rs1045642* and *rs4148738* shows increased peak levels of dabigatran.

The *ABCB1* gene is not only responsible for variations in the drug response of dabigatran but rivaroxaban and apixaban as well, but at different loci. *rs1045642* and *rs4148738* SNPs in *ABC1* are responsible for increased drug levels of rivaroxaban and *rs4148738* in the same gene is responsible for increased drug levels of apixaban [67, 81]. There is scope for more studies on the pharmacogenomics of the newer DOACs however, the literature is limited.

With all the extensive research done on pharmacogenomics, especially warfarin, it is now time to consider using genotype-focused warfarin algorithms. However, since most of the studies were focused on Europeans, it would be wise to implement these algorithms after conducting studies that included more diverse populations [82].

### 2.5 Diuretics

Diuretics have proven to be useful in treating conditions such as heart failure, hypertension, and clinical congestion [83, 84]. The goal of their treatment is to achieve euvolemia [82], in other words, a state in which the body would have a normal volume of blood or fluids. Overall, the diuretics work by ensuring the balance of water and sodium in the body by reabsorbing the sodium in the kidney. There are two commonly used types of diuretics: thiazides, which are inhibitors of sodium chloride cotransporter, and loop diuretics, which, by targeting the sodium potassium chloride co-transporter regulate sodium in the kidney. Furthermore, some diuretics act on the sodium channel ENac, which are called potassium-sparring diuretics [85]. The illustration depicts the proposed mechanism of action of both thiazide [83] and loop diuretics [84]. Fig. (**5**) demonstrates the mechanism of action of thiazides and loop diuretics.

A report from the Joint National Committee on Prevention, Detection, Evaluation, and Treatment of High Blood Pressure states that thiazide diuretics are first ranked when it comes to hypertensive treatments. Diuretics help patients reach their desired blood pressure (BP). The adducin gene, a cytoskeletal gene containing α and β subunits encoded by *ADD1, ADD2,* and *ADD3,* has been the interest of multiple studies. The α-adducin gene, *ADD1*, is associated with thiazide in patients treated for hypertension. More specifically, researchers have been investigating *SNP Glyn460Trp* in *ADD1* polymorphism which is associated with salt-sensitive hypertension [49]. The antihypertensive response to hydrochlorothiazide was linked to the polymorphism Gly460 [84]. Another conducted study has contradicted the statements that *ADD1* and *Gly460* treat hypertension, stating that they are connected to a high risk of myocardial infarction (MI) or stroke during treatment [86]. Furthermore, multiple studies indicated that the *Trp460* variant plays a role in helping reduce BP and lowering the risk of an MI or a stroke when treated with hydrochlorothiazide compared to chlorthalidone [49]. More specifically, compared to the individuals with the *Gly460* genotype, individuals with the *460Trp* variant have an increased protective effect of about 38% against MIs and strokes [87]. The atrial natriuretic precursor A (*NPPA*) has evolved in the world of pharmacogenomics. It is essential to withdraw the atrial peptide (ANP), which acts as a diuretic by regulating extracellular fluid volume and electrolyte homeostasis [49]. Research suggested associations between NPPA and diuretics [87]. More specifically, the *NPPA rs5065 (T2238C)* variant showed C allele carriers to have lower risks of cardiovascular diseases (CVD) and, more importantly, BP reductions with the use of chlorthalidone compared to amlodipine. However, chlorthalidone has been associated with higher risks of heart attack or stroke in patients with the TT genotype [87]. Also, an association has been determined between G polymorphisms and diuretics. More precisely, the G protein β 3 subunit *C825T* polymorphism has been found to have an antihypertensive response to thiazide diuretics. The study has indicated a decline of 10/6 mmHg of average BP in CC homozygotes, 14/8 mmHg in CT heterozygotes, and 16/11 mmHg in TT homozygotes. Moreover, a mean decline of BP of 15 mmHg in Trp460 allele carriers was determined compared to a 7-mmHg decline in the *Gly460* homozygotes [83].

Further on, consistent links have been found between genetic variations in the *NEDD4L* and hypertension treatment with thiazide diuretics [87, 88]. The *NEDD4L* removes the epithelial sodium channel, a fine-tuning mechanism for sodium excretion in the kidney, from the cell surface by encoding regulatory proteins. Multiple knock-out mouse studies have determined higher levels of ENac expressions, salt-sensitive hypertension, and responsiveness to amiloride in mouse analog *NEDD4L* knock-outs. In humans, *NEDD4L,* more precisely *rs4149601*, is linked with salt-sensitive hypertension and response to thiazide diuretics. The splicing of the G allele that is led by the A>G polymorphism demonstrates increased levels of BP, salt sensitivity, and decreased plasma renin activity. Based on these results, a hypothesis could be made that a greater BP response to thiazide diuretics should be observed in the G carriers if the G allele contributes to salt-sensitive diuretics [88]. A study conducted by the genome-wide association (GWA) found interesting links between chromosome 12q15 and *FRS2*, as well as thiazide diuretics [49]. The study was conducted on 194 Black individuals and 195 white individuals with a range of good and bad BP. In primary genome scans, variations in *12q15* in Black people were found to be significantly associated with BP response. Additionally, potential candidate genes that could influence BP response to hydrochlorothiazide were determined in a haplotype analysis of 35 tag single nucleotide polymorphisms (SNP) in the 3 genes, which emerged to exclude *FRS2* and to favor *YEATS4* over *LYZ* [89]. Another study has demonstrated that a BP response to hydrochlorothiazide is influenced by chromosome *17q24* variation within *PRKCA* in white people of European descent. That study also determined that chromosome 20q13.32 variation in the region between *GNAS* and *EDN3* might influence BP response to hydrochlorothiazide as well [90].

Loop diuretics are usually used for the relief of symptoms when water and sodium are retained. Some recent studies have found that the excretion amounts of sodium, chloride, potassium, and calcium were impacted by polymorphisms in *GNB2, ANP, ACE,* and *ADD1* in response to loop diuretics [91]. Another study has found that IL-6 levels decreased in patients using furosemide. More specifically, an increased risk for the development of heart failure (HF) was determined in individuals with variations in the G allele of *rs1800795* on the *IL-6* gene. In addition, researchers determined an association between -572G/C (rs1800796) mutation occurring in the *IL-6* gene and cardiovascular diseases, coronary heart diseases, and abdominal aortic aneurysms. Furthermore, studies have found that the *UGT1A1* gene, which encodes UGT enzymes and prevents the accumulation of toxic waste in our bodies through glucuronidation, can trigger the development of HF. *UGT1A1*’s variations, more precisely *rs88729* and *rs4148323*, have been found to be resistant to HF drug therapies. Researchers investigated allelic discrimination of *UGT1A1* and IL-6 variations in three groups: responders, non-responders, and control group. When comparing the results of the *UGT1A1* in the three groups, 55 T-insertion was observed to be statistically high in the non-responder group. Overall, it was concluded that furosemide resistance may be caused by *rs1800796* mutation in *IL-6* and by *55 T*-insertion in the *UGT1A1* [92].

### 2.6 Calcium Channels Blockers

Hypertension and cardiovascular diseases are leading causes of morbidity and mortality worldwide that present a significant public health challenge [93]. Calcium channel blockers (CCBs) play a crucial role in managing these conditions by inhibiting the entry of calcium ions into the heart and blood vessel walls, which leads to vasodilation and reduced blood pressure [94].

#### 2.6.1 Mechanism of Action

Calcium channel blockers (CCBs) inhibit the L-type calcium channels on vascular smooth muscle and cardiac myocytes, reducing the influx of calcium ions essential for muscle contraction. This action results in vasodilation of peripheral arteries and reduced myocardial force generation, thereby decreasing heart rate and lowering blood pressure [95]. The effectiveness of CCBs can be significantly influenced by genetic variability in the receptors they target. For example, variations in the *CACNA1C* gene, which encodes the alpha-1C subunit of the L-type calcium channel, can affect the response to CCBs, necessitating personalized medication selection to optimize therapeutic outcomes. CCBs are categorized into dihydropyridines, which predominantly act on vascular smooth muscle, leading to vasodilation with minimal effect on cardiac contractility, and non-dihydropyridines, which have a more pronounced effect on the heart, reducing heart rate and contractility [94]. This dual classification highlights their custom application in treating hypertension, angina, and arrhythmias. It further emphasizes the versatility and specificity of CCBs in cardiovascular therapy.

#### 2.6.2 Clinical Applications

Calcium channel blockers (CCBs) are versatile medications used in the treatment of various cardiovascular conditions. They are primarily used to manage hypertension by effectively lowering blood pressure and decreasing the risk of stroke and heart disease and also play a crucial role in treating angina pectoris by alleviating chest pain due to reduced blood flow to the heart muscles, while certain non-dihydropyridine CCBs are employed in controlling specific types of arrhythmias, aiding in the normalization of heart rhythms [96, 97]. The effectiveness of CCBs varies across different patient populations as it is influenced by factors such as age, race, and comorbid conditions [98]. For example, dihydropyridine CCBs are often more effective in older patients and those of African descent for managing hypertension [94].

#### 2.6.3 Pharmacogenomics of Calcium Channel Blockers

The field of pharmacogenomics explores how genetic variations affect an individual’s response to drugs, including calcium channel blockers (CCBs). Research has identified specific genetic markers that can predict the efficacy and side effects of CCBs in treating hypertension and cardiovascular diseases [99, 100]. Variations in genes that encode for the calcium channels themselves, such as *CACNA1C,* or genes involved in their metabolic pathways, such as *CYP3A4* and *CYP3A5*, can significantly influence how an individual metabolizes and responds to CCBs. For instance, mutations in the *CACNA1C* gene can alter the functionality of L-type calcium channels, affecting the efficacy of CCBs targeting these channels. Similarly, variations in the *CYP3A4* and *CYP3A5* genes can affect the metabolism of CCBs, influencing their blood levels and therapeutic effects. Recognizing these genetic differences is essential for healthcare providers to adopt a personalized medicine approach, allowing for the tailoring of treatment plans based on each patient’s unique genetic makeup. This approach not only enhances the efficacy of CCB therapy but also minimizes the risk of adverse drug reactions, optimizing patient care and outcomes in hypertension, angina pectoris, and arrhythmias management [101]. This maximizes the therapeutic effectiveness of CCBs along with minimizing the risk of adverse reactions, heralding a new era in precision cardiovascular care.

Recent gene studies have revealed the advances in the mechanism of gene mutations as an etiology to hypersensitivity to CCBs as well as in shaping the response to them, which contribute to a better personalized cardiovascular therapy [99]. One such study was conducted to determine the influence of polymorphisms in the *CACNACI* gene, which codes for the alpha1C subunit of L-type calcium channel on CCB effectivity [95]. Data provided the evidence that specific *CACNA1C* gene variants had effects on the drug's sensitivity, therefore some variants increased the drug potency in causing blood pressure reduction, while others did not, which designated the genetic background for recommendations for drug dosage and duration [95]. Moreover, investigations have been made on the relationship between genetic variations of responsible genes of drug metabolism pathways, especially *CYP3A4* and *CYP3A5* genes coding the cytochrome P450 enzymes, and pharmacokinetics and treatment outcomes of CCBs [99]. Genetic variants in enzymatic activity of CCBs can have a strong effect on drug metabolism and change concentrations and RBCs bioavailability. As a result, drugs may prove inefficacious or cause more side effects in the carriers of particular SNPs, highlighting thus the need for personalized dosing strategies that take into consideration SNPs and other genetic factors. In addition to that, genetic studies identified the relationship between gene polymorphisms and the risk of CCB-related side effects, *e.g.*, peripheral edema or gingival hyperplasia [99]. Through genomic studies, variants in calcium genes and genes responsible for cell signaling pathways have been pointed out as factors that may complicate people’s medical treatment [95]. This information can be used to formulate stratified patient groups and risk mitigation approaches. The implementation of genetic data into clinical practice by providers facilitates the organization of CCB treatment regimens in a way that individual profiles allow clinical professionals to maximize treatment efficacy while minimizing side effects. This precision drug treatment approach will, undoubtedly, drastically improve the way cardiovascular care is currently managed since it allows medical professionals to individualize treatment plans and boost patients’ health in situations like hypertension, angina pectoris, and arrhythmias.

### 2.7 Antiarrhythmic Drugs

Antiarrhythmic medications, targeted at reestablishing and preserving normal heart rhythm, are a fundamental component of the treatment of cardiac arrhythmias. These medications are essential for relieving symptoms, lowering morbidity, and averting potentially fatal arrhythmia-related consequences [102]. Antiarrhythmic drugs are indispensable in stabilizing supraventricular tachyarrhythmias and potentially fatal ventricular arrhythmias [103]. These pharmacological medicines function to restore normal heart rhythm by selectively targeting certain ion channels and myocardial substrates [104]. For healthcare providers to maximize treatment outcomes, it is essential to understand their mode of action.

#### 2.7.1 Mechanism of Action

The cardiac action potential is an ion movement cycle that causes the cardiac myocyte to successively depolarize and repolarize, which causes the muscle to contract [105]. In a nutshell, the antiarrhythmic drugs interfere with ion movement across different stages of the cardiac action potential. The mechanism of action of various classes of antiarrhythmic drugs is illustrated in Fig. (**6**) [106].

#### 2.7.2 Pharmacogenomics

Comprehending the pharmacological characteristics of antiarrhythmic medications makes it imperative for physicians to personalize treatment plans and improve therapeutic outcomes. For the individualized treatment of cardiac arrhythmias, it is essential to comprehend the pharmacogenomic parameters that affect the drug’s safety, effectiveness, and tolerance.

The most common genetically driven adverse effect of antiarrhythmics is drug-induced QT prolongation. Roden *et al.* [107] stated that genes encoding drug-metabolizing enzymes, including cytochrome P450 (CYP) enzymes, have genetic variations that affect the safety and effectiveness of antiarrhythmic medications. Changes in the *CYP2D6* gene may have an impact on the way class I antiarrhythmics, such as propafenone and flecainide, are metabolized. Poor metabolizer phenotypes may be associated with elevated plasma levels of procainamide, which predisposes to side effects such QT prolongation and drug-induced lupus syndrome [107]. The effectiveness of currently available ion channel blocking medications in treating cardiac arrhythmias has been unsatisfactory, and medications that have undergone testing have not been able to reduce mortality [108, 109]. Drug targets like cardiac ion channels can be impacted by genetic variations, which can potentially impact a drug's efficacy and susceptibility to side effects. The response to flecainide therapy has been linked to genetic differences in the *SCN5A* gene, which codes for the cardiac sodium channel Nav1.5. The effectiveness of the medication and the risk of proarrhythmia can both be impacted by specific mutations in *SCN5A* that result in gain- or loss-of-function mutations in the sodium channel [110]. In another study, the effectiveness of anti-arrhythmic medications has been observed to be impacted by mutations in the Na and K channels. The affinity of these medicines for voltage-gated sodium channels is affected by mutations and polymorphisms in these channels [111]. Certain genetic polymorphisms increase the risk of adverse drug reactions to antiarrhythmics. The response to sotalol and dofetilide therapy has been associated with genetic variations in the *KCNH2* gene, which codes for the heart potassium channel hERG (Kv11.1). Kannankeril *et al.* [112] found that the drug's effectiveness and the likelihood of Torsades De Pointes can be affected by mutations that change the hERG channel's binding affinity and repolarization kinetics [112].

There is a paucity of studies done to understand the clinically relevant genetic variations, advances in pharmacogenomics, and technology. Further studies are needed to maximize antiarrhythmic therapy effectiveness and enhance patient outcomes.

### 2.8 Statins

Statins are molecules of fungal origin discovered in 1973 by Akira Endo and his co-workers in Japan [113].

The first statin, compactin or mevastatin, was isolated from Penicillium citrinum [113]. Lovastatin, the first commercial statin to be approved by the FDA was independently discovered by two scientists in the year 1976 [113, 114]. As of today, there are only 7 statins approved by the FDA for clinical use, which include Atorvastatin, Rosuvastatin, Simvastatin, Pravastatin, Fluvastatin, Lovastatin, and Pitavastatin [115].

The FDA-approved indications of statins include: 1. Hyperlipidemia and dyslipidemia, 2. atherosclerosis, 3. primary prevention of atherosclerotic cardiovascular disease (ASCVD), 4. secondary prevention in patients with clinical ASCVD, and 5. Pediatric and adult patients with familial hypercholesterolemia [115].

Statins target hepatocytes and work by inhibiting hydroxymethylglutaryl-CoA (HMG-CoA) reductase (HMGR) enzyme that converts HMG-CoA into mevalonic acid, a cholesterol precursor and work by reversible competitive inhibition [116, 117]. Statins not only compete with the normal substrate of HMG-CoA reductase, but they also alter the binding site and prevent it from attaining a functional structure [116]. By inhibiting the synthesis of cholesterol, statins upregulated hepatic low-density lipoprotein receptor (LDL) receptors and increased the clearance of LDL-cholesterol (LDL-C) [118]. Fig. (**7**) demonstrates the mechanism of action of statins.

A deeper understanding of cholesterol biosynthesis is essential to study the pharmacogenomic variations of statins. Not all patients prescribed with statins have the same therapeutic benefits and some patients are even more at a higher risk of developing adverse reactions to statins. Current research states that individual response to statins is dependent on many genetic factors like gene polymorphisms and non-genetic factors such as age, sex, smoking status, comorbid diabetes, and ethnicity [119].

#### 2.8.1 SLC Transporters

*OTP1B1* and *OTP2B1* encoded by *SLCO1B1* and *SCLO2B1* are influx transporters that play a crucial role in statin uptake by the hepatocytes, where they undergo a first pass metabolism [120-124]. The first pass metabolism in the liver may decrease the systemic availability of statins, but it is more beneficial since hepatocytes and their role in cholesterol biosynthesis is the most desired primary effect of statins [124]. SLCO1B1*5 and SLCO1B*15 are variants of the gene variants *SLCO1B1* that have currently been studied and proven to impact the pharmacokinetic properties of statins [122, 123]. Kameyama *et al.* mentioned that the alleles SLCO1B1*5 and SLCO1B*15 had significantly decreased the exposure to Pravastatin, Atorvastatin, and Cerivastatin [123]. Another study on the SLCO1B1*5 allele revealed the effect on simvastatin; no effect *in vitro* but 2 times increased exposure to Simvastatin compared to non-carriers [125]. Existing literature also has information revealing the decrease in exposure to Atorvastatin, Pitavastatin and Rosuvastatin in homozygous carriers [126]. Another variant of *SLCO1B1* is SLCO1B1*1B which showed a 65% reduction in the area under the curve for pravastatin concentration but an increase in the plasma levels for another drug in the class, Simvastatin [127, 128]. Even though this allele altered the plasma concentration of drugs, no significant effects were identified in terms of LDL reduction, which is the main desired effect of the drug [129].

#### 2.8.2 ABC Genes

Dietary fatty acids are absorbed by intestinal cells *via* ATP binding cassette proteins encoded by genes like *ABCB1, ABCG2,* and *ABCG8* [10, 122, 124, 130]. MDR1 encoded by *ABCB1,* is an efflux transporter that is responsible for the biliary and urinary elimination of statins [10]. Polymorphisms in these genes have been proven to influence the lipid lowering properties of statins. Most common variants of the *ABCB1* gene include *c.1236C>T (rs1128503),* c.2677G>T/A (*rs2032582*) and c.3435C>T (*rs1045642*) [10]. Fiegenbaum *et al.* found that the presence of *ABCB1C.12367* gene allele had a better reduction in cholesterol levels compared to the wilder allele [131]. A different study by Rebecchi *et al.* showed that the presence of *2677T* allele showed a better decrease in LDL-c levels and apolipoprotein B levels in response to atorvastatin [132]. Individuals carrying the APOE ε2 allele, compared to those with the ε4 allele, experienced a notably more significant reduction in LDL-C levels when treated with atorvastatin or pravastatin and were more likely to reach the guideline-recommended target of LDL-C ≤70 mg/dL. Additionally, polymorphisms in the triallelic G2677T/A variant of the ABCB1 gene were linked to the extent of LDL-C reduction with pravastatin therapy [133].

#### 2.8.3 Cytochrome P 450 System (CYP)

Statins are metabolized by the cytochrome P 450 (CYP) system in the liver. Most statins are metabolized by *CYP3A4/5* isoenzymes with the exception of Fluvastatin and rosuvastatin, which are metabolized by *CYP2C9* and Pitavastatin which is excreted unchanged by the kidneys [122, 134]. Research reveals that genetic alterations in *CYP3A* are 20-40-fold more common than any other genetic variation, making it the most important candidate for pharmacogenetic investigations [13]. A study conducted by Becker *et al.* stated that the presence of variant allele CYP3A4*1B has proven to lower the risk of elevated plasma levels of atorvastatin, simvastatin and lower risk of dose decrease during therapy compared to those having the wild gene [135]. On the other hand, the presence of CYPA5*3 variant allele resulted in low or non-expression of *CYP3 A5,* leading to significantly lower metabolism of atorvastatin, lovastatin and simvastatin compared to expression [136]. Finally, genetic variations due to missense mutations of *CYP2C9* leading to the formation of two variant alleles CYP2C9*2 and CYP2C9*3, significantly lower the enzyme activity and decrease the metabolism of statins with CYP2C9*3 having much dangerous effect in terms of developing adverse reactions [137].

#### 2.8.4 Cholesterol Metabolism Genes

HMGR enzyme catalyzes the rate limiting step of cholesterol biosynthesis in the liver and is the main target of statins (118,119,124,126). The gene *HMGCR* encodes for HMGR and there are 2 variants of this gene *HMGCR rs17244841 (g.14863A>T)* and *rs3846662 (c.1564-106A>G)* that were proven by several independent scientists to have a decreased effect of statins in patients with dyslipidemia and familial hypercholesterolemia [122, 130, 138, 139]. Other important proteins that should be studied to have an enhanced understanding of pharmacogenomics are LDL receptor (LDLR), apo B and proprotein convertase subtilisin/kexin type 9 (*PCSK9*), all proven to have variants that could alter the response to statins [122]. There are two variants studied by independent scientists that are known to decrease the effects of statins in patients: *HMGCR rs 17244841* [130, 138, 139]. A genome-wide study conducted on European patients found out about an *LDLR* variant *LDLR rs688 (c.1773C>T, p.Asn591=)* that was responsible for a decreased response to statin (lower reduction in LDL cholesterol reduction) [140]. PCSK9 is a protease enzyme that interacts with LDLR and directs them to lysosomes for degradation, because of which lesser LDL receptors are recruited on the cell surface, leading to a decrease in intracellular LDL levels and an increase in plasma LDL levels [122, 140]. This physiology led to a few more studies that concluded that loss of function mutation in *PSCK9* gene led to a better response to statins and gain of function mutation had the opposite effect [122, 141, 142].

#### 2.8.5 Pleiotropic Effects of Statins

Landmark clinical trials on statins concluded that statins can be used as first line agents for reducing the total blood cholesterol level and LDL-c [10]. Statins have additional effects on the human body, which are independent of their LDL-c reducing properties. These auxiliary cholesterol-independent effects are called pleiotropic effects [143-145]. Existing literature suggests that a lot of genes and their variants could be contributing to these effects. Some of the important pleiotropic effects of statins in cardiovascular disease (CVD) are its antioxidant effects, and effects on vascular smooth muscle cell proliferation. Endothelial nitric oxide synthase (eNOS), which is encoded by the gene *NOS 3* is responsible for vascular smooth muscle cell proliferation [118, 122, 146]. A study conducted in a group of healthy Brazilian males revealed that a variant of the *NOS 3* gene *rs2070744* (g.6933C>T) was associated with a decrease in triglyceride levels in response to short term treatment with atorvastatin [122, 146]. Recent studies have implicated the role of the manganese-dependent superoxide dismutase enzyme (SOD) in the development of CVD [147]. A study conducted on Brazilian cohorts revealed that a particular genotype of *SOD 2, rs4880* CC showed lower total and LDL cholesterol reduction and low HDL cholesterol increase after treatment with rosuvastatin [122, 148].

#### 2.8.6 Adverse Effects of Statins

Adverse drug reactions of statins include the commonly reported statin related muscle symptoms (SAMS) and less frequently reported statin induced liver toxicity and central nervous system toxicity [10, 122]. SAMS is reported to be the most common cause of statin therapy non-adherence and discontinuation [122, 149, 150].

Existing literature states that the extent of these adverse reactions can also be dependent on the presence or absence of certain variants of genes or mutations or gene polymorphisms responsible for the pharmacokinetic and pharmacodynamic properties of statins. According to a study by Turner *et al.*, [150] the one most important gene responsible for SAMS is a variant of the transport protein *SLCO1B1 rs4149056* [150].

### 2.9 Challenges and Limitations of Pharmacogenomics

Pharmacogenomics is a personalized medicine therapy that maximizes therapeutic benefits and reduces side effects based on patient's genetic composition. Incorporating pharmacogenomics into clinical practice presents an array of challenges despite its conceivable advantages. The majority of cardiovascular disorders are polygenic. The intricate nature of genetic interactions in cardiovascular diseases and the wide range of individual drug responses present a significant challenge [151]. Numerous genetic variations are still poorly known or need more extensive research to validate their correlations with drug response, even if some of them have long-standing links. Additionally, most pharmacogenomic research has focused on single-gene drug interactions, not considering the intricate relationships between several genetic variations and environmental factors that together affect drug response [11]. Drug–drug or drug–diet interactions can affect the effects of various medications. Pharmacogenomic studies are challenging to control because food and drug interactions can affect a drug's efficacy [152]. With the increasing trend of multimorbidity [153], the intake of multiple drugs has also increased, which is leading to drug-drug-gene interactions [154]. This may present as a direct interaction of drugs causing induction, inactivation of one of the drugs, or both. The interactions may also cause a gain of function or loss of function of the genes responsible for the metabolism of the drugs [154].

The response of a drug is not solely dependent on its metabolism but is a multifactorial process [155]. This may include age, sex [156], HLA [155], and even the microbiota of the individual [8]. Not enough studies have been conducted to see the change in pharmacogenomics in relation to the other factors affecting drug responses.

The pharmacogenomic studies conducted on the drug responses in CVD have been mainly focused on the European population. More studies focused on pharmacogenomic variations of drug responses in other races must be conducted to start targeted therapy [76, 157]. Personalized treatment using pharmacogenomics may cause bias in the treatment of individuals. Screening of all the individuals based on race may cause stereotyping of the patients and in some cases, screening may be completely unnecessary as the gene described for a particular race may be absent in the population hailing from a particular geographical area [158].

The main challenge facing the clinical application of genetic biomarker testing is that there are few of these tests that possess the necessary sensitivity, specificity, and predictive value to be effectively employed as screening instruments for predicting therapeutic efficacy and averting adverse drug reactions [152]. To effectively interpret genetic data and transform it into actionable treatment recommendations, pharmacogenomic-guided therapy requires interdisciplinary collaboration involving clinicians, genetic experts, and pharmacists. This demonstrates the importance of comprehensive training programmes in preparing medical providers to successfully integrate pharmacogenomics into clinical decision-making [159].

Regulations and enforcement challenges impede the application of pharmacogenomic discoveries in clinical practice. Before licensing pharmacogenomic tests for general use, regulatory bodies like the European Medicines Agency (EMA) and the U.S. Food and Drug Administration (FDA) require strong proof of clinical validity and utility. Large-scale prospective trials, longitudinal follow-up, and real-world data are frequently needed to demonstrate the clinical value of genetic biomarkers in predicting drug response. These processes can be time- and resource-intensive [160]. Furthermore, as novel data about the relationship of genetic variations to medication response and disease susceptibility becomes available, their clinical importance may change over time [161]. Based on continuous research and clinical trials, pharmacogenomic guidelines and recommendations may need to be modified on a regular basis. On the other spectrum of things, not just administration but also patients require strong proof and the need to accept the need for personalized medicine and practices [162].

Pharmacogenomics in the cardiovascular system has to contend with substantial technical hurdles due to limits in genetic sequencing and data interpretation. Genetic test interpretation can be complicated by false-positive and false-negative results, as well as variations of unknown significance (VUS), which can result in treatment recommendations that are not optimal [159].

A number of ethical and legal concerns come up as pharmacogenomics is incorporated into therapeutic practice. These include patient privacy concerns, the need for informed permission for genetic testing, and the possibility of genetic discrimination by employers or insurance based on genetic data. The ethical application of personalized medicine in cardiovascular care necessitates respecting patient autonomy and privacy as well as providing fair access to pharmacogenomic testing [163].

Another huge factor that needs to be considered is the cost of the testing and implementation of such personalized treatment plans for individuals. Although some studies show the cost-effectiveness of pharmacogenomic testing prior to starting the treatment [164]. The studies are limited to drugs like clopidogrel and warfarin [165]. The cost-effectiveness may also vary depending on the number of genes tested [166]. Sometimes there may be multiple genes for a single drug, and testing all of them may be a costly endeavor for many. The cost of testing may vary from place to place; what might be a cost-effective test for individuals in the USA may not be cost-effective for other developing countries [166].

### 2.10 Future Directions

The constant quest for precision medicine in the treatment of cardiovascular diseases (CVDs) is bringing pharmacogenomics into the limelight. Potential breakthroughs have been made in pharmacogenomics through persistent exploration. But, there remain some areas where further studies can build on existing data.

To improve the accuracy of treatment response and prognosis prediction, future research needs to give precedence to recognizing and verifying the presence of innovative pharmacogenomic biomarkers that are unique to cardiovascular disorders [167]. Targeted therapies with improved risk prediction are made possible by the integration of genomic risk scores, which incorporate various genetic variations linked to CVDs [168]. Polygenic risk scores have been demonstrated to identify individuals who are more susceptible to cardiovascular events, which could enable early treatments and preventive measures [169]. An advancement in incorporating pharmacogenomics in CVDs might stem from additional research to enhance these genomics risk scores.

In order to guarantee widespread acceptance among a variety of patient populations, future initiatives should concentrate on enhancing the availability and cost-effectiveness of genetic testing for cardiovascular diseases [170]. Engaging healthcare consumers, implementation scientists, and health economists in performing outcome studies and creating payment plans that will encourage pharmacogenomic testing. Providing patients with thorough knowledge about pharmacogenomics and how it affects cardiovascular care can encourage proactive engagement in personalized healthcare, compliance with treatment, and well-informed decision-making [171]. Healthcare professionals can make real-time therapeutic decisions by customizing treatment plans based on each patient's genetic profile when pharmacogenomic data is incorporated and available in EHRs [161]. Continuing research on this could lead to more precise, effective, and patient-centered cardiovascular care.

## CONCLUSION

The global search continues for safer and more effective treatment options for cardiovascular disorders, which continue to be the leading cause of death. Adoption of pharmacogenomics as a clinical approach may radically transform the way we manage cardiovascular diseases. Determining an individual’s genetic profile can identify patients who might benefit from different medicines and dose adjustments or who are more likely to experience adverse effects and help us formulate personalized therapy. Although pharmacogenomics is an advanced technique, utilizing it for personalized therapy still requires a better understanding of the differences and variations among the population. Genotyping and lack of evidence-based studies are major obstacles to implementing pharmacogenomics. The pharmacogenomics of only certain drugs has been studied extensively and the implications of polymorbidity on the genetic variations need to be considered. Hence, continuous research and clinical diligence are required to bring about the utilization of pharmacogenomics in personalized therapy.

## Figures and Tables

**Fig. (1):**
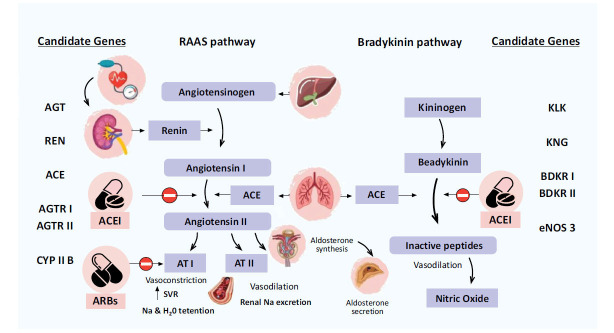
Mechanism of action of ACEI and ARB with underlying pathophysiology.

**Fig. (2):**
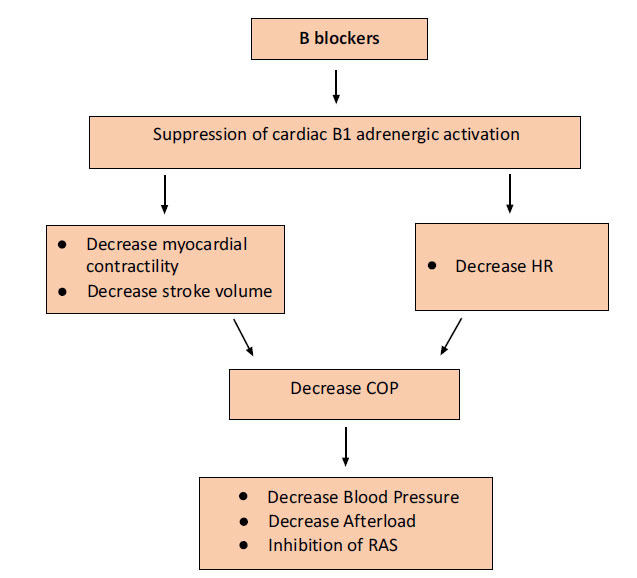
Mechanism of action of β blockers [43].

**Fig. (3):**
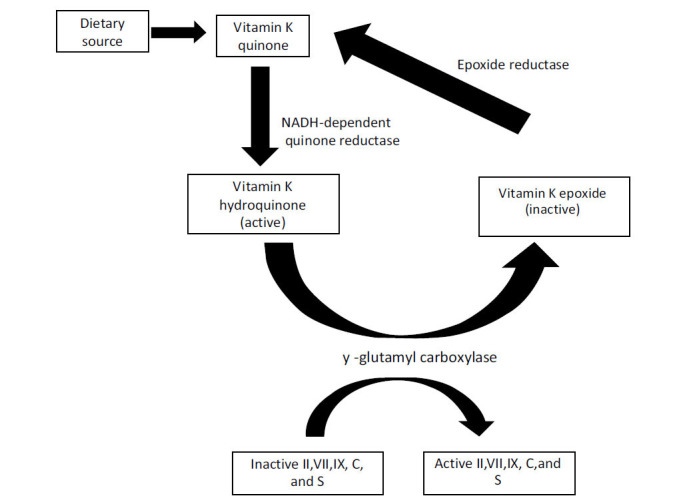
Mechanism of action of vitamin k [60].

**Fig. (4):**
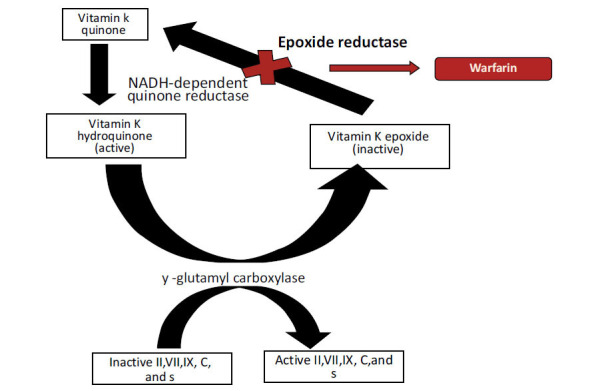
Mechanism of action of warfarin .

**Fig. (5):**
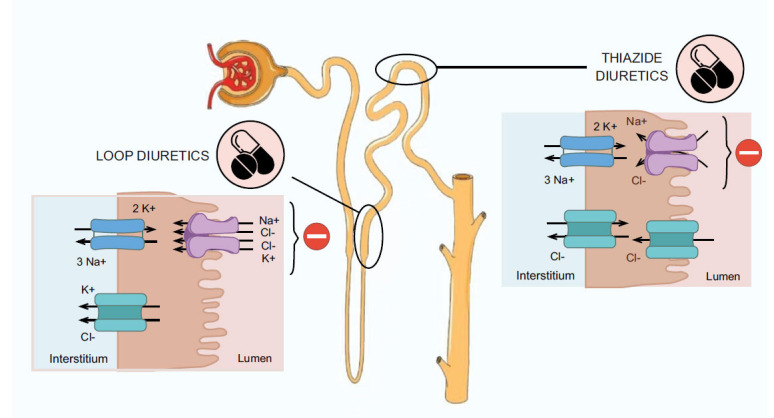
Mechanism of action of thiazides and loop diuretics.

**Fig. (6):**
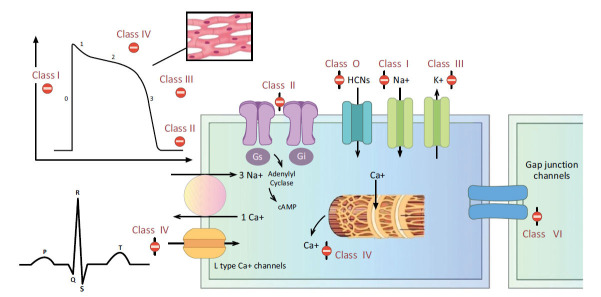
Mechanism of action of antiarrhythmic drugs.

**Fig. (7):**
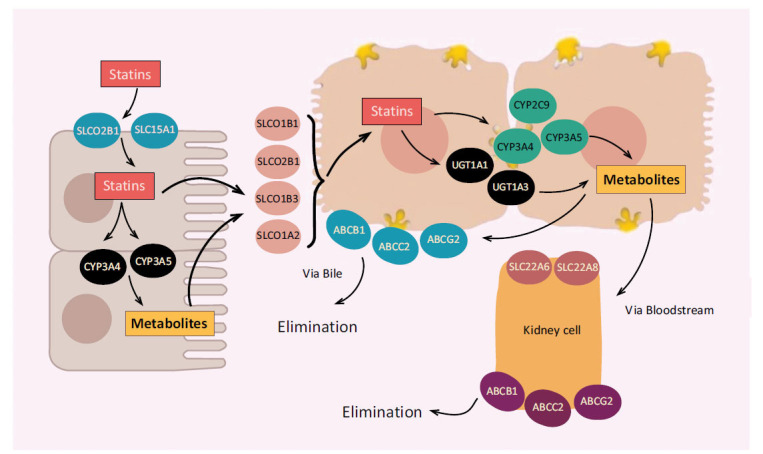
Mechanism of action of statins.

**Table 1:** Responses to ACE inhibitors and ARB modulated by the presence of pleomorphisms in different genes.

Gene	Polymorphism	Association with ACEI/ ARB Response
***ACE***	Intron 16 (*rs1799752*) Insertion/ deletion (I/D)	D allele: improved hypertensive control with ACEI [26]
		I allele: better hypertensive control than D allele [26]
		D allele: decreased serum levels of *ACE* [25]
***AGT***	SNP Met235Thr (*rs699*)	No significant impact on ACEI and ARB response [28]
***CYP11b2***	SNP −344C/T (*rs17998*)	Altered hypertensive outcomes and aldosterone levels with C allele [32]
		With T allele in SILVHIA trial [33]
***AGTR1***	SNP (rs275651) (*rs5182*)	Genetic risk profiling in patients with stable coronary artery disease – PERGENE [37]
***BDKR 1***	(*rs12050217*)	
***eNOS 3***	−786T/C (*rs2070744*)	C allele: enhanced ability to generate NO in treatment with ARB olmesartan [40]
	−665C/T SNP (*rs3918226*)	Associated with better responses to enalapril [41]
***eNOS 3***	TC genotype (*rs2070744*) SNP	The coexistence of these genetic variations is associated with an improved response to enalapril therapy [42]
***BDKRB2***	CC genotype (*rs1799722*) SNP	

## LIST OF ABBREVIATIONS

ACEI: Angiotensin Converting Enzyme Inhibitor
AHA/ACC: American Heart Association/American College of Cardiology
ARBs: Angiotensin Receptor Blocker
CPIC: Clinical Pharmacogenetics Implementation Consortium
CVD: Cardiovascular Diseases
CVDs: Cardiovascular Disorders
PCI: Percutaneous Coronary Intervention
